# Exploring Vaccine Hesitancy Among Parents in a Tribal Community: A Cross-Sectional Study in Ormanjhi, Jharkhand

**DOI:** 10.7759/cureus.87362

**Published:** 2025-07-05

**Authors:** Dewesh Kumar, Manish Shekhar, Nusrat Noor, Shashi B Singh, Ankita Mukul, Apoorva Wasnik

**Affiliations:** 1 Community Medicine/Preventive and Social Medicine, Rajendra Institute of Medical Sciences, Ranchi, IND; 2 Epidemiology, Integrated Health Information Platform, Deoghar, IND; 3 General Practice, Clinica Cure Hospital, Ranchi, IND

**Keywords:** immunization, parents’ awareness, tribal health, vaccination coverage, vaccine hesitancy

## Abstract

Background: Vaccine hesitancy poses a significant challenge to immunization programs, particularly in tribal communities where unique sociocultural and systemic barriers exist. This study aimed to assess the prevalence, determinants, and underlying reasons for vaccine hesitancy among parents of children under seven years of age in Ormanjhi, a tribal block in Jharkhand, India.

Methods: A community-based cross-sectional study was conducted from April to September 2017 using the World Health Organization (WHO) cluster sampling method to select 30 clusters and 210 households. Data were collected using a pretested, semi-structured questionnaire covering sociodemographic characteristics, Vaccine Appropriate for Age (VAFA), and reasons for vaccine hesitancy. Descriptive statistics, agreement analysis, and chi-square tests were applied for data analysis.

Results: Vaccine hesitancy was reported by 15.24% (n = 32) of parents, while only 22.9% (n = 48) of children were vaccinated appropriately for their age. The most common reasons for hesitancy included fear of adverse effects (8.57%), lack of knowledge (6.19%), and mistrust (6.19%). Hesitancy was significantly associated with mothers as the primary responders (p = 0.036) and beliefs in alternative preventive methods (p = 0.019). Other sociodemographic factors, such as education and caste, showed no significant association.

Conclusion: In Ormanji, vaccine hesitancy is largely driven by misinformation, fear, and mistrust, compounded by low vaccination coverage. Targeted interventions, particularly community-based education and trust-building through local health workers, are essential to improving immunization rates in tribal communities.

## Introduction

Vaccine hesitancy, defined as the delay in acceptance or refusal of vaccines despite their availability, is a complex and multifaceted public health challenge [1,2]. It encompasses a spectrum of attitudes, ranging from complete acceptance to outright refusal, with intermediate levels of doubt or partial compliance. The phenomenon is driven by an interplay of factors, including misinformation, distrust in healthcare systems, cultural beliefs, and perceived risks outweighing benefits [3,4]. The advent of social media has amplified these concerns, spread unfounded fears, and eroded public confidence in vaccines [5-7]. Historical medical injustices, particularly in marginalized communities, have further entrenched skepticism toward healthcare institutions [8]. Understanding and addressing vaccine hesitancy are critical to achieving optimal vaccination coverage and controlling vaccine-preventable diseases.

Globally, vaccine hesitancy has been recognized as a significant barrier to immunization programs, with the World Health Organization (WHO) listing it among the top 10 global health threats in 2019 [3,9,10]. Prevalence varies widely, with studies reporting hesitancy rates ranging from 10% to 30% across different populations [11,12]. High-income countries often cite safety concerns and distrust in pharmaceutical industries, while low- and middle-income countries face additional challenges like access barriers and misinformation [5,7]. For instance, hesitancy toward the human papillomavirus (HPV) vaccine has been notable in regions with strong cultural or religious objections [10]. The global resurgence of diseases like measles underscores the urgent need to address this issue [3].

In India, vaccine hesitancy is a growing concern, particularly in rural and tribal areas where healthcare access and awareness are limited [13]. National surveys indicate that approximately 20%-25% of parents express some level of hesitancy, with higher rates for newer vaccines like HPV and rotavirus [2,9]. Misinformation, religious beliefs, and distrust in government health programs contribute significantly [7]. Regional variations exist, with lower vaccination coverage in northern and eastern states compared to southern states.

Jharkhand, a predominantly tribal state in eastern India, faces unique challenges in vaccine uptake. Tribal communities, which constitute about 26% of the state’s population, often exhibit higher hesitancy due to cultural beliefs, limited healthcare infrastructure, and historical mistrust in medical interventions [8]. In areas like the Ormanjhi, a block in Ranchi district, logistical barriers and low health literacy exacerbate the issue, making it a critical region for studying vaccine hesitancy.

Despite the global and national focus on vaccine hesitancy, there is a paucity of research in India’s tribal regions, particularly in Jharkhand, where sociocultural and systemic factors create unique challenges [13]. Understanding the specific drivers of hesitancy in these communities is essential for designing targeted interventions. Therefore, this study aims to investigate the prevalence, determinants, and reasons for vaccine hesitancy among parents of children under seven years of age in Ormanjhi block, Ranchi district, Jharkhand, to inform evidence-based strategies for improving vaccination coverage in tribal communities.

## Materials and methods

Study design and setting

This community-based cross-sectional study was conducted from April to September 2017 in the Ormanjhi block of Ranchi district, Jharkhand, a predominantly tribal area with a population of 105,623. The block includes 27 subcenters and is characterized by limited healthcare infrastructure. The study was facilitated by the Rural Health and Training Centre (RHTC) under the Department of Community Medicine, Rajendra Institute of Medical Sciences, Ranchi.

Sampling technique

The WHO 30 x 7 sampling method was employed [14], which estimates immunization coverage within ±10% at a 95% confidence level. A two-stage sampling process was used. First, 30 clusters were selected using probability proportionate to population size, with each subcenter considered a cluster. Three subcenters, namely, Chakla, Irba, and Siladri, were each divided into two clusters due to their high population coverage compared to other centers. In the second stage, seven subjects were selected per cluster by identifying a random starting household and selecting one eligible child per household. Sampling continued with the nearest household until seven children were enrolled in each cluster.

Eligibility criteria

Parents were included if they could provide documentation of their child's vaccination history from birth up to seven years of age and gave informed consent to participate. Children were excluded if they had specific health conditions that limited routine immunization or if they were recent immigrants to the locality (residing in the area for less than one year) from other blocks or districts.

Data collection

Data were collected over a two-month period using a self-designed, face-validated, and pretested semi-structured questionnaire, developed based on established vaccine hesitancy tools and indicators. The questionnaire included sections on sociodemographic characteristics, VAFA status according to the national immunization schedule, and reasons for hesitancy. Interviews were conducted by trained staff, with rapport established through support from personnel at the RHTC.

Data management and analysis

Data were compiled in Microsoft Excel (Microsoft Corp., Redmond, WA, US) and analyzed with Jamovi version 2.6.23. Quantitative variables (e.g., age, VAFA) were assessed using Student’s t-test after verifying normality assumptions. Qualitative variables (e.g., reasons for hesitancy) were analyzed using the chi-square test. Agreement analysis was performed using Wilson confidence intervals for proportions to assess parental attitudes and beliefs.

To compare vaccine-related attitudes between parents of children with VAFA and those expressing vaccine hesitancy, chi-square tests of association were conducted across 14 binary-response survey items (yes/no/don’t know). These items evaluated dimensions such as vaccine awareness, trust, perceived necessity, and safety concerns. The chi-square test assessed the null hypothesis of no association between respondent group (VAFA vs. hesitant) and survey responses, with statistical significance set at p < 0.05. All expected cell frequencies were verified to meet the chi-square assumption (≥5 per cell).

Study variables

VAFA was defined as having received all age-appropriate vaccines according to India’s National Immunization Schedule (NIS) under the Universal Immunization Programme (UIP) by the age of seven years. The recommended vaccines include the following: at birth, Bacillus Calmette-Guérin (BCG), oral polio vaccine (OPV)-0, and hepatitis B; at 6, 10, and 14 weeks, pentavalent (diphtheria, pertussis, tetanus (DPT), hepatitis B (Hep B), *Haemophilus influenzae* type b (Hib)), OPV, rotavirus (in select states), and fractional inactivated poliovirus vaccine (fIPV) (at 6 and 14 weeks); at 9 months, measles, Japanese encephalitis (JE) (in endemic areas), and vitamin A; at 16-24 months, measles-2, JE-2, diphtheria, pertussis, tetanus (DPT), and OPV boosters; and at 5-6 years, diphtheria, pertussis, and tetanus (DPT) booster. Children not receiving the full schedule by age seven were classified as non-compliant or not vaccinated appropriately.

Vaccine hesitancy was assessed through self-reported reluctance or delay in accepting vaccines, along with stated reasons such as fear of adverse effects, mistrust, and lack of awareness. Sociodemographic variables included age, sex, religion, caste, education level, occupation, and family type.

Ethical considerations

Ethical approval for the study was obtained from the Institutional Ethics Committee of Rajendra Institute of Medical Sciences (Memo No. 118 IEC/IRC, dated February 15, 2017). Informed consent was obtained from all participants prior to data collection. Confidentiality was strictly maintained, and all data were used solely for research purposes.

## Results

Prevalence of vaccine hesitancy and VAFA

Of the 210 households surveyed, 15.24% (n = 32) of parents reported vaccine hesitancy, 83.81% (n = 176) were non-hesitant, and 0.95% (n = 2) were unsure. Only 22.9% (n = 48) of children were vaccinated appropriately for age, while 77.1% (n = 162) were not fully vaccinated as per the NIS.

Sociodemographic characteristics

Table 1 presents a comparison of demographic characteristics between children with and without VAFA. Children with VAFA were significantly older on average (mean age 40.7 months, SD 20.2) compared to those without VAFA (mean age 30.1 months, SD 24.8; p = 0.008). No significant differences were observed in birth order, birth interval, number of siblings, or respondent age. VAFA status did not significantly vary by sex, religion, caste, ethnicity, type of delivery, parental education or occupation, or family type (p > 0.05).

**Table 1:** Sociodemographic characteristics of the participants ^1^Independent sample t-test. ^2^Chi-square test of independence. VAFA: Vaccine Appropriate for Age.

VAFA		No	Yes	Total	Test statistic	p-value
Total N (%)	N (%)	162 (77.1)	48 (22.9)	210		
Age (months)	Mean (SD)	30.1 (24.8)	40.7 (20.2)	32.5 (24.2)	-3.022^1^	0.008
Birth order	Mean (SD)	1.9 (1.1)	1.7 (1.0)	1.9 (1.1)	1.189^1^	0.139
Birth interval (years)	Mean (SD)	3.2 (0.8)	3.1 (0.9)	3.2 (0.9)	0.693^1^	0.683
Siblings	Mean (SD)	2.2 (1.1)	2.2 (1.0)	2.2 (1.1)	0.116^1^	0.867
Respondent age (years)	Mean (SD)	26.9 (5.8)	26.8 (5.2)	26.8 (5.6)	0.114^1^	0.897
Sex	Female (%)	79 (48.8)	19 (39.6)	98 (46.7)	1.255^2^	0.339
	Male (%)	83 (51.2)	29 (60.4)	112 (53.3)		
Religion	Christian (%)	2 (1.2)	0 (0.0)	2 (1.0)	1.938^2^	0.586
	Hindu (%)	82 (50.6)	24 (50.0)	106 (50.5)		
	Muslim (%)	56 (34.6)	20 (41.7)	76 (36.2)		
	Sarna (%)	22 (13.6)	4 (8.3)	26 (12.4)		
Caste	General (%)	22 (13.6)	13 (27.1)	35 (16.7)	6.190^2^	0.101
	Other backward caste (%)	90 (55.6)	26 (54.2)	116 (55.2)		
	Scheduled caste (%)	3 (1.9)	1 (2.1)	4 (1.9)		
	Scheduled tribe (%)	47 (29.0)	8 (16.7)	55 (26.2)		
Ethnicity	Non-tribal (%)	114 (70.4)	40 (83.3)	154 (73.3)	2.74^2^	0.110
	Tribal (%)	48 (29.6)	8 (16.7)	56 (26.7)		
Delivery	Institutional (%)	141 (87.0)	43 (89.6)	184 (87.6)	0.049^2^	0.825
	Non-institutional (%)	21 (13.0)	5 (10.4)	26 (12.4)		
Mother's education	Illiterate (%)	31 (19.1)	5 (10.4)	36 (17.1)	7.048^2^	0.070
	Primary school (%)	19 (11.7)	2 (4.2)	21 (10.0)		
	Middle school (%)	44 (27.2)	21 (43.8)	65 (31.0)		
	Higher secondary and above (%)	68 (42.0)	20 (41.7)	88 (41.9)		
Mother's occupation	Housewife (%)	129 (79.6)	39 (81.2)	168 (80.0)	2.298^2^	0.512
	Daily wage worker (%)	9 (5.6)	2 (4.2)	11 (5.2)		
	Part-time farming in own field (%)	18 (11.1)	7 (14.6)	25 (11.9)		
	Private job (%)	6 (3.7)	0 (0.0)	6 (2.9)		
Father occupation	Unemployed (%)	8 (5.0)	1 (2.1)	9 (4.3)	2.379^2^	0.662
	Farming (%)	24 (15.0)	9 (19.1)	33 (15.9)		
	Shopkeeper (%)	16 (10.0)	4 (8.5)	20 (9.7)		
	Daily wage worker (%)	66 (41.2)	15 (31.9)	81 (39.1)		
	Government job (%)	4 (2.5)	1 (2.1)	5 (2.4)		
	Private job (%)	42 (26.2)	17 (36.2)	59 (28.5)		
Father's education	Illiterate (%)	17 (10.6)	4 (8.5)	21 (10.1)	2.963^2^	0.394
	Primary school (%)	20 (12.5)	2 (4.3)	22 (10.6)		
	Middle school (%)	67 (41.9)	22 (46.8)	89 (43.0)		
	Higher secondary and above (%)	56 (35.0)	19 (40.4)	75 (36.2)		
Family	Nuclear (%)	66 (40.7)	18 (37.5)	84 (40.0)	0.054^2^	0.814
	Joint (%)	96 (59.3)	30 (62.5)	126 (60.0)		
Respondent	Mother (%)	145 (89.5)	37 (77.1)	182 (86.7)	6.608^2^	0.036
	Father (%)	14 (8.6)	7 (14.6)	21 (10.0)		
	Others (%)	3 (1.9)	4 (8.3)	7 (3.3)		

Reasons for hesitancy

Table 2 outlines the reported reasons for vaccine hesitancy among parents. The most common concerns included fear of adverse effects (8.57%, n = 18), lack of knowledge or information (6.19%, n = 13), doubts about vaccine efficacy (6.19%, n = 13), and mistrust in general (6.19%, n = 13). Less frequently cited reasons included a low perceived severity of vaccine-preventable diseases (1.90%, n = 4) and a preference for alternative methods of prevention (3.33%, n = 7).

**Table 2:** Reasons for vaccine hesitancy among parents of children under seven years of age

Variable	Not applicable N (%)	Yes N (%)	No N (%)	Don't know N (%)
Hesitant	-	32 (15.24)	176 (83.81)	2 (0.95)
Reasons for hesitancy				
Low perception of vulnerability/severity of the disease	200 (95.24)	4 (1.90)	5 (2.38)	1 (0.48)
Fear of adverse effects	182 (86.67)	18 (8.57)	5 (2.38)	5 (2.38)
Doubts about vaccines	191 (90.95)	13 (6.19)	6 (2.86)	-
Influence of information on vaccine	198 (94.29)	11 (5.24)	1 (0.48)	-
Mistrust in general	195 (92.86)	13 (6.19)	2 (0.95)	-
Preference of other modes of prevention	199 (94.76)	7 (3.33)	3 (1.43)	1 (0.48)
Lack of knowledge/information	195 (92.86)	13 (6.19)	2 (0.95)	-
Others	208 (99.05)	-	2 (0.95)	-

Attitudes and beliefs

Hesitant parents were significantly less likely to trust vaccine-related information (88% agreement vs. 98% in non-hesitant, p = 0.001), perceive vaccine-preventable diseases as severe (88% vs. 90%, p = 0.001), or feel comfortable discussing vaccinations with healthcare providers (66% vs. 88%, p = 0.001). They were also more likely to believe that a healthy lifestyle (50% vs. 23%, p = 0.001) or alternative medicines (38% vs. 16%, p=0.002) could serve as substitutes for vaccines. Fear of vaccination was significantly more prevalent among hesitant parents (63% vs. 14%, p = 0.001), as shown in Table 3.

**Table 3:** Percentage agreement between VAFA parents and hesitant parents VAFA: Vaccine Appropriate for Age.

		VAFA		Hesitant	
Items	Total agreement, % agreement (95% CI)	Agreement in with VAFA, % agreement (95% CI)	Agreement in without VAFA, % agreement (95% CI)	Agreement in hesitant parents, % agreement (95% CI)	Agreement in non-hesitant parents, % agreement (95% CI)
Do you trust the information about the vaccine you received?	96 (93.3, 98.7)	94 (91, 97)	96 (93, 99)	88 (83, 92)	98 (96, 100)
Do you think diseases can be prevented by vaccines?	93 (89.5, 96.5)	98 (96, 100)	91 (87, 95)	91 (87, 95)	94 (91, 97)
Do you think the diseases prevented by vaccines are serious?	89 (84.8, 93.2)	94 (91, 97)	88 (83, 92)	88 (83, 92)	90 (85, 93)
Are you comfortable asking any doctor or nurse about vaccines?	83 (77.9, 88.1)	88 (83, 92)	82 (77, 87)	66 (59, 72)	88 (83, 92)
Do you think a good lifestyle like eating healthy foods can eliminate the need for vaccination?	27 (21, 33)	13 (8.5, 18)	31 (25, 37)	50 (43, 57)	23 (18, 29)
Do you think the need for vaccination can be eliminated by relying on alternative medicines, like chiropractic, homeopathy, or naturopathy?	19 (13.7, 24.3)	15 (10, 20)	20 (15, 25)	38 (32, 45)	16 (12, 22)
Do you think children today are given too many vaccines?	42 (35.3, 48.7)	29 (23, 35)	46 (39, 53)	59 (52, 65)	39 (33, 46)
Do you think the immune system gets weakened by vaccination?	18 (12.8, 23.2)	15 (10, 20)	19 (14, 25)	31 (25, 37)	15 (10, 20)
Are you afraid of being vaccinated?	22 (16.4, 27.6)	10 (5.9, 14)	25 (20, 31)	63 (56, 69)	14 (10, 19)
Do people around you hesitate from having their children vaccinated?	24 (18.2, 29.8)	21 (15, 27)	25 (20, 31)	34 (28, 41)	22 (17, 28)
Do you think it is important to vaccinate your child to prevent the spread of diseases?	88 (83.6, 92.4)	88 (83, 92)	88 (83, 92)	84 (78, 88)	89 (84, 93)
Do you feel pressured by people close to you or from society to have your child vaccinated?	36 (29.5, 42.5)	35 (29, 41)	36 (30, 43)	56 (49, 63)	32 (26, 39)

Association between VAFA and hesitancy

Table 4 summarizes the chi-square tests assessing associations between 14 questions. Of the 14 questions analyzed, 10 showed statistically significant associations (p < 0.05), indicating notable differences in vaccine-related attitudes between parents of VAFA children and those identified as hesitant. In particular, significant associations were observed for the question, “Are you afraid of being vaccinated?” (p < 0.001), with hesitant parents more likely to report fear. Other significant associations included trust in vaccine information (p = 0.036), belief in the severity of vaccine-preventable diseases (p = 0.031), and comfort in discussing vaccination with healthcare providers (p = 0.007).

**Table 4:** Association between VAFA and hesitant parents with the questionnaire items VAFA: Vaccine Appropriate for Age.

Items	VAFA	Hesitant	χ^2^ statistic	p-value
Are you aware of the vaccines a child has to get?	108	108	3.139	0.076
Has your child received all the vaccines proposed?	145	145	5.756	0.016
Do you trust the information about the vaccine you received?	201	200	4.654	0.037
Do you think diseases can be prevented by vaccines?	195	195	4.665	0.031
Do you think the diseases prevented by vaccines are serious?	187	187	4.518	0.033
Are you comfortable asking any doctor or nurse about vaccines?	175	175	7.168	0.007
Do you think a good lifestyle like eating healthy foods can eliminate the need for vaccination?	57	56	7.208	0.007
Do you think he need for vaccination can be eliminated by relying on alternative medicines, like chiropractic, homeopathy, or naturopathy?	40	40	2.983	0.084
Do you think children today are given too many vaccines?	89	88	1.345	0.246
Do you think the immune system gets weakened by vaccination?	38	37	1.496	0.212
Are you afraid of being vaccinated?	46	45	14.996	<0.001
Do people around you hesitate from having their child vaccinated?	50	50	0.010	0.919
Do you think it is important to vaccinate your child to prevent the spread of diseases?	184	184	4.863	0.027
Do you feel pressured by people close to you or from society to have your child vaccinated?	75	74	0.093	0.760

## Discussion

This study in the Ormanjhi block, a tribal area in Jharkhand, found a vaccine hesitancy prevalence of 15.24%, which is lower than some global estimates ranging from 20% to 30% in certain populations [11,12]. However, the high proportion of children not vaccinated appropriately for their age highlights a significant public health concern, where vaccine hesitancy often overlaps with challenges in access to healthcare services [13].

Our findings reveal clear differences in vaccine-related attitudes between VAFA and hesitant parents. Hesitant parents showed significantly lower trust in vaccine information (p = 0.037), were less likely to believe vaccines prevent serious diseases (p = 0.031), and felt less comfortable discussing vaccination with doctors or nurses (p = 0.07). Many also believed a healthy lifestyle could replace the need for vaccines, a belief held by only 13% of VAFA parents, reflecting cultural and traditional influences previously noted in other studies [11].

Certain beliefs, such as reliance on alternative medicines, concern over the number of vaccines, or fears that vaccines weaken the immune system, did not differ between groups. This may be due to limited responses for some questions or shared community beliefs across both groups. Similarly, peer hesitancy and feelings of pressure to vaccinate did not show strong associations, suggesting these may be less influential factors in this setting.

Unlike findings in other studies where parental education or caste have a notable impact on vaccine uptake [10], these variables were not significantly associated with hesitancy in Ormanjhi. This may be due to relatively homogeneous beliefs and practices within the tribal community. Interestingly, mothers, who were more frequently the respondents, showed greater hesitancy, possibly due to greater exposure to community myths and misinformation as primary caregivers [7].

Overall, fear, mistrust, and lack of knowledge emerged as the main drivers of vaccine hesitancy, exacerbated by limited access to healthcare facilities in the region [8]. To improve vaccination rates, targeted community-based interventions are essential. Strategies such as training local health workers (e.g., Accredited Social Health Activists (ASHAs)) to address fears and misinformation, engaging trusted tribal leaders, and deploying mobile vaccination units to remote areas have shown success in similar contexts [9].

Limitations

This study has several limitations. Its cross-sectional design prevents any inference of causality, and the relatively small sample size (n = 210) may limit the generalizability of the findings. Vaccine hesitancy was self-reported and may be influenced by social desirability bias. The effects of factors such as age, education, and occupation were not thoroughly explored, further limiting generalizability. Additionally, potential confounders like distance to vaccination centers and access to healthcare services were not assessed, which may have introduced bias. Future research should consider longitudinal designs with larger and more diverse populations to better understand temporal trends and regional differences.

## Conclusions

The study highlights a prevalence of vaccine hesitancy of 15.24% among parents in Ormanjhi, Jharkhand, with 77.1% of children not vaccinated appropriately for their age. Key drivers of hesitancy included fear of adverse effects, lack of knowledge, and mistrust in the healthcare system, challenges commonly observed in tribal communities. The significant association between hesitancy and mothers as respondents underscores the need for gender-specific interventions. Additionally, the inverse relationship between VAFA and belief in lifestyle-based prevention points to the role of cultural perceptions in shaping vaccine attitudes. Unlike prior studies, sociodemographic factors such as education did not significantly predict hesitancy, suggesting that systemic barriers and widespread misinformation may play a more influential role in this setting. To address these issues, we recommend community-based strategies such as training ASHA workers to counter myths, involving tribal leaders to build trust, and deploying mobile vaccination camps to improve access. Future research should explore longitudinal patterns and gather qualitative insights into cultural and historical mistrust to inform targeted interventions. Strengthening vaccination coverage in Ormanjhi and similar tribal regions is essential for reducing vaccine-preventable diseases and promoting equitable health outcomes.

## Appendices

Questionnaire

A.      Sociodemographic Characteristics

1. Name of the child:

2. Date of birth:

    Age:

3. Sex: 

    1. Male

    2. Female

4. Religion:

    1. Hindu

    2. Muslim      

    3. Sikh            

    4. Christian    

    5. Sarna         

    6. Others

5. Caste:

    1. General      

    2. OBC                       

    3. SC              

    4. ST

6. Ethnicity:

    1. Tribal               

    2. Non-tribal

7. Birth order:  

   1. First     

   2. Second     

   3. Third     

   4. Fourth     

   5. Fifth or higher

8. Birth interval:

    1. One year                    

    2. Two years       

    3. Three or more years.

    4. NA

9. Number of siblings:

    1. None       

    2. One     

    3. Two     

    4. Three or more   

10. Delivery:

      1. Institutional                 

      2. Home

11. Mother’s education:

     1. Illiterate      

     2. Primary      

     3. Middle       

     4. Higher secondary and above

12. Mother’s occupation:

      1. Housewife          

      2. Daily wage worker

      3. Part-time farming in own field                    

      4. Government job         

      5. Private job

13. Father’s education:

      1. Illiterate        

      2. Primary      

      3. Middle       

      4. Higher secondary and above

14. Father’s occupation:

      1. Unemployed           

      2. Farming     

      3. Shopkeeper

      4. Daily wage worker  

      5. Government job    

      6. Private job

15. Socioeconomic status: Total family income / total members =

16. Family:

     1. Joint                     

     2. Nuclear

17. Respondent:

     1. Mother        

     2. Father         

     3. Other (relationship with the child:)

18. Respondent's age (years):

B. Immunization History

1. Immunization card:

   1. Yes              

   2. No

2. Immunization table (as per record or respondent’s recall) (Table 5)

**Table 5:** Vaccines administered

Vaccines	Received			Date received	Appropriate age			Delay (in days)
	Yes	No	Not applicable		Yes	No	Not applicable	
BCG								
Hep B_0_								
OPV_0_								
Penta 1/DPT1								
Penta 2/DPT2								
Penta 3/ DPT3								
OPV1								
OPV2								
OPV3								
IPV								
Measles 1								
JE-1								
Measles 2								
JE-2								
DPT B1/OPV B1								
DPT B2								
Any other vaccine								

3. Does your child receive OPV doses on SIA days?

    1. Yes (always)          

    2. Sometimes       

    3. No

4. BCG scar present:

    1. Yes                

    2. No

5. Other vaccines received other than those used in EPI:

   1.

   2.

6. History of side effects:

C.    Vaccine Hesitancy

**Table 6:** Vaccine hesitancy

S. no.	Items	Yes		No		Don’t know
1.	Are you aware of the vaccines a child has to get?					
2.	Has your child received all the vaccines proposed?					
		Strongly agree	Somewhat agree	Somewhat disagree	Strongly disagree	
3.	Are you hesitant to have your child vaccinated?					
	Reasons:					
	Low perception of vulnerability/severity of the disease					
	Fear of adverse effects					
	Doubts about vaccines					
	Influence of information on vaccine					
	Mistrust in general					
	Preference for other modes of prevention					
	Lack of knowledge/information					
	Other					
4.	Do you trust the information about the vaccine you received?					
5.	Do you think diseases can be prevented by vaccines?					
6.	Do you think the diseases prevented by vaccines are serious?					
7.	Are you comfortable asking any doctor or nurse about vaccines?					
8.	Do you think a good lifestyle like eating healthy foods can eliminate the need for vaccination?					
9.	Do you think the need for vaccination can be eliminated by relying on alternative medicines, like chiropractic, homeopathy, or naturopathy?					
10.	Do you think children today are given too many vaccines?					
11.	Do you think the immune system gets weakened by vaccination?					
12.	Are you afraid of being vaccinated?					
13.	Do people around you hesitate from having their child vaccinated?					
14.	Do you think it is important to have your child vaccinated to prevent the spread of diseases?					
15.	Do you feel pressured by people close to you or from society to have your child vaccinated?					
